# Research knowledge transfer to improve the care and support of adolescents with sickle cell disease in Ghana

**DOI:** 10.1111/hex.13573

**Published:** 2022-07-31

**Authors:** Brenda A. Poku, Alison Pilnick

**Affiliations:** ^1^ School of Sociology and Social Policy University of Nottingham Nottingham UK

**Keywords:** adolescents, fatigue, Ghana, knowledge transfer, research dissemination, sickle cell disease, young people

## Abstract

**Introduction:**

Effective transfer of research findings to key knowledge users, particularly in low‐ and middle‐income countries, is not always achieved, despite being a shared priority among researchers, funders, healthcare and community stakeholders and decision‐makers. A constructivist grounded theory study conducted in 2015–2019 in Ghana that explored sickle cell‐related fatigue in adolescence resulted in numerous implications for practice and policy. Peer‐reviewed funding was obtained to support disseminating these findings to relevant stakeholders.

**Methods:**

Key steps in implementing this study dissemination project included: (1) identifying and attracting target stakeholders from healthcare and community organizations; (2) tailoring tools for communication of research findings for the stakeholder groups and (3) designing interactive workshops to facilitate knowledge sharing and uptake.

**Finding:**

Despite the COVID‐19 pandemic, 50 healthcare and community stakeholders participated in the dissemination workshops. The dissemination activities contributed new layers of understanding to the original research findings through discussions. Through the workshops, participants identified culturally valuable and actionable recommendations that they could take forward to improve care and support for young people with sickle cell disease in Ghana. A follow‐up 6 months post the workshops indicated some positive knowledge usage and benefits.

**Conclusion:**

This dissemination project provided a unique opportunity for researchers and stakeholders to share in the interpretation of research findings and to strategically plan recommendations to improve SCD‐focused care and support for young people in Ghana. Further research dissemination should continue to be grounded in locally generated knowledge, include systematic, long‐term evaluation of dissemination outcomes and be adequately financed.

**Patient and Public Contribution:**

Public involvement in this study was critical to the research dissemination project. The Sickle Cell Association of Ghana (Kumasi chapter) actively supported the project's development, organization and facilitation. Parent members of the Association, the Association's executive members and volunteers, and the health professionals involved in sickle cell care at the Komfo Anokye Teaching Hospital participated in the project workshops. They contributed to the knowledge transfer and uptake.

## 1 INTRODUCTION

Evidence‐based policy and practice can lead to improved health and development outcomes, more efficient use of limited resources, and greater accountability.^1^ However, a significant gap exists between health research and policy and practice, despite the increasing demand to close this ‘know‐do’ gap through knowledge transfer and exchange.^1^, ^2^ The gap is larger in low‐ and middle‐income countries (LMICs), given the inherent challenges related to research equity globally.^2^ Although new knowledge, when well used, has the potential to save lives and improve welfare in LMICs,^3^ health research capacity in LMICs is mainly insufficient. It tends to be funded by donors from high‐income countries (HICs) who typically require HIC researchers to lead research projects.^4^, ^5^ These compromise the extent to which research aligns with LMIC's research priorities, is perceived as locally relevant and credible by key stakeholders, and is ultimately taken up in policy and practice.^4^, ^5^, ^6^ Consequently, there are limited published examples of effective knowledge transfer and exchange work in LMICs, and limited knowledge and understanding of what types of activities encourage and support knowledge transfer practice.^2^ This paper addresses this knowledge gap by describing a research dissemination project carried out in Ghana.

Research utilization does not begin until systematic communication of new and existing knowledge occurs.^7^ Conventional research dissemination approaches such as journal publications are increasingly under critique for reinforcing passive, top–down relationships among producers and users of knowledge and assuming that simple receipt of relevant information will lead to change in practice.^8^ Strategies for maximizing the dissemination of research findings include (1) use of theoretically guided principles to communicate findings broadly, proactively and interactively^7^ using a variety of information‐sharing techniques; (2) providing knowledge users with opportunities to discuss and interpret research findings within their local context^9^ and (3) addressing different information needs and social contexts of all knowledge users who need to collaborate to implement research findings.^10^, ^11^ In addition, multifaceted interactive educational interventions are identified as particularly effective dissemination strategies.^12^ This article describes a project that used the above strategies to disseminate pertinent sickle cell disease (SCD)‐focused research findings to healthcare and community stakeholders.

SCD is the most common genetic blood disorder worldwide, predominant in sub‐Saharan Africa, where an estimated 1000 babies are born with the condition daily.^13^ It is a chronic debilitating condition commonly characterized by chronic anaemia and fatigue and unpredictable and episodic acute painful episodes or crises that cause cumulative damage to multiple organ systems. In Africa, 50%–90% of children born with SCD die prematurely before their fifth birthday due to delayed diagnosis and suboptimal management of SCD and their related complications, while those who survive become vulnerable to the exacerbations of the condition and its implications.^13^, ^14^ Indeed, it is estimated that SCD contributes 9% of the mortality rate in children under 5 years in the West African subregion and 16% in some West African countries, including Ghana.^15^ However, studies exploring the impact of providing evidence‐based SCD education to key stakeholders/knowledge users in Africa are absent. Specific strategies for disseminating SCD‐focused research findings to broader and more varied audiences have not yet been reported in the literature. The identification of stakeholders, development of content and format for this dissemination project were guided by three key components related to effective knowledge transfer: *cognitive* (i.e., sharing knowledge based on the research findings), *experiential* (i.e., linking the information conveyed to personal experiences) and *practical* (i.e., ensuring the inclusion of key stakeholders who contribute to care and support).^7^

## 2 METHODS AND MATERIALS

This dissemination work was conducted as part of the first author's Economic and Social Research Council postdoctoral fellowship. However, the study forming the basis of the work was undertaken in 2015–2019 as part of the first author's doctoral research. This used constructivist grounded theory to explore the fatigue experiences of adolescents with SCD in Ghana.^16^ The study involved 24 adolescents with SCD (aged 12–17 years) recruited from the teaching hospitals in Ghana's two main cosmopolitan cities and employed semi‐structured interviews, drawing, photography and picture‐elicitation interviews to generate data.^17^ The study findings highlighted several critical issues related to the meaning and significance of fatigue for adolescents with SCD in Ghana and the care and support they received. These included (1) fatigue having a significant negative impact on the young people's everyday life and representing the most restrictive and disruptive aspect of growing up with SCD^16^; (2) fatigue representing a socially undesirable feature that was stigmatizing and, therefore, a significant threat to ‘normalcy’; (3) fatigue having significant actual and potential consequences for the adolescents' biographies in adolescence and adulthood^18^; (4) gender differences in fatigue experience and parental care and support^19^; (5) absence of fatigue‐related support and care from healthcare professionals^18^ and (6) despite the established biobehavioural model of SCD‐related fatigue,^20^ there were salient social factors that influenced the symptom experience among young people.^16^, ^18^, ^19^

Despite the chronicity and biopsychosocial significance of SCD‐related fatigue,^19^, ^21^, ^22^, ^23^, ^24^, ^25^, ^26^ it tends to be an ‘orphaned’ symptom scarcely prioritized in research and clinical care compared to SCD‐related pain.^20^, ^22^, ^26^ This lack of attention has been associated with the nihilistic perception that fatigue is an inevitable consequence of the condition, about which nothing can be done and therefore must be endured by those living with SCD.^27^ The six main findings from the research study are, therefore, particularly significant in a context where fatigue is treated as an ‘orphan’ symptom. The challenge of disseminating our findings to reinforce the legitimacy and significance of fatigue and encourage attention to it in care, support and research required strategic considerations of the target audience, the information to prioritize for dissemination and the format and structure of the dissemination work to maximize transfer and uptake of this new knowledge.

### 2.1 The target audience

Improving the care and support of children and young people (CYP) with SCD, particularly in Ghana, requires the concerted efforts of a community of stakeholders, including governmental health, educational and social institutions, care providers (e.g., physicians, nurses, psychologists, educators, pharmacists, physical therapists), families (parents, carers, siblings, extended relatives), support groups and charities (health, health‐related and educational) and community and religious leaders.^28^ Given the limited funding to conduct this dissemination project, target audiences had to be strategically selected to focus on those who had an immediate need for the knowledge and play a central role in care and support provision, as well as key change agents and opinion leaders.^29^ We capitalized on an opportunity to collaborate and partner with two long‐standing SCD‐focused charities in Ghana to align the project more closely with their priorities and practice. These charities, playing pivotal roles in developing the best services for patients, are vital components of comprehensive programmes in Ghana. They are staffed by individuals with SCD and their families, key healthcare professionals and individual supporters from the wider community.

Consultations took place with the charities' leads early in the project development regarding which stakeholders to target and how best to reach them to enhance the project's outcomes. The charities were also involved as collaborators in the funding application. The initial project aim was to work with them to facilitate workshops to engage approximately 40 parents/carers, 30 healthcare professionals (from the SCD healthcare teams in the two research settings) and 10 of the charities' leads/volunteers with the key research findings. We planned to conduct four workshops in the two cities where the original study was conducted. However, we experienced several challenges to the initial plan postfunding award. First, we lost contact with one of the charities despite their active involvement during the project development and funding application. Several attempts to contact them postaward and discuss the project in light of the COVID‐19 pandemic were futile. Second, the COVID‐19 pandemic and the national travel restrictions and bans in both the United Kingdom and Ghana threatened the feasibility of the dissemination work. Third, there were uncertainties regarding the available charity's (the Sickle Cell Association of Ghana [SCAGH]) capacity to support the work during a pandemic. Lastly, even when the dissemination work was feasible, the continued risks of COVID‐19 and the precautionary measures needed to mitigate these meant additional funds were required, which had not been included in the initial funding application.

As a result, the planned dissemination work was downsized. Efforts were redirected to replanning the workshops within the funding limits, the SCAGH's reduced support capacity, and COVID‐19 restrictions, protocols and preventive measures. In consultation with SCAGH, dissemination was focused on two workshops in the city where most of the study population was recruited. We agreed on one workshop for 20 parents of CYP with SCD and another comprising 15 healthcare professionals (from the SCD healthcare team in one of the research settings) and 5 SCAGH leads/volunteers. On recommendation from the charity and clinical partners, a senior SCD medical doctor and two SCAGH leads were included in the parents' workshop to provide immediate support regarding the feasibility of any clinical‐ and social‐specific comments, reactions and ideas from participating parents.

### 2.2 Format of the dissemination work

In light of the COVID‐19 pandemic and travel restrictions, the research team, SCAGH and clinical partners considered remote facilitation of the workshops. However, we concluded that remote facilitation would not be the most appropriate mitigation for the face‐to‐face workshops due to the following concerns:

1. There was a potential limitation of workshop participants' access to computers and the internet and the erratic nature of internet delivery and connection in Ghana. This would have required SCAGH to bring participants together physically to enable participation with only the workshop lead (first author) being distant.
2. Taking the workshops online would rely solely on SCAGH, who were already experiencing increased workload due to their communities' increased demand for support due to the pandemic. Adding the burden of time and effort to organize and facilitate additional events was not acceptable. It would also have caused further delay to the delivery of the engagement activities, in addition to that caused by the COVID‐19 lockdown measures in the United Kingdom and Ghana.
3. Moving the dissemination activities online would have posed a challenge for evaluating the impact of the activities. SCAGH were concerned that the workshops with parents/carers should include those with diverse literacy capabilities. Therefore, a paper‐based or computer‐based evaluation would have been impractical.

Given these concerns, SCAGH and the clinical partners recommended that the dissemination work take the form of face‐to‐face as initially planned, but in line with the United Kingdom and Ghana governments' COVID‐19 protocols and measures (masking, social distancing, hand hygiene and adequate ventilation) to ensure safer participation for all the stakeholders. Consequently, the first author travelled to Ghana to organize and lead the work with support from our partners.

Although the dissemination project activities were designed to be COVID‐19 safe and offered free of charge to the participants, additional strategies were developed to encourage and increase attendance. For instance, on the recommendation of SCAGH and the clinical partners, the original plan of holding half‐day workshops to share the study findings was pared down to two and a half‐hour sessions. Rather than create a separate meeting to add to everyone's full agenda, permission was obtained from both SCAGH and the SCD healthcare team to use one of their regularly scheduled meetings for the workshops. The healthcare professionals' workshop was integrated into one of the SCD healthcare teams' weekly clinical meetings and took place in one of the hospital's conference rooms. For the parents' workshop, we capitalized on the World Sickle Cell Awareness Day commemoration by SCAGH and integrated the activities into their planned activities. Snacks, lunch and other refreshments were offered to workshop participants at the project's expense. Participants were provided with an additional incentive of £25: £10 to defray travel costs and £15 as a ‘thank you’ for their attendance. Plans were also made to provide on‐site childcare for participants who needed it during the workshops.

### 2.3 Dissemination tools and activities

Although the content for this dissemination project was selected in consideration of the divergent needs and interests of the two sets of specific stakeholders targeted for dissemination, these were grounded in the same research findings. Connecting the knowledge with the stakeholders' specific priorities and practices was more likely to impact their opinions and decision‐making.^30^ However, as the parents' workshop was held first, ideas and recommendations from this were subsequently included in the healthcare professional and charity lead/volunteer workshop (see Table 1).

**Table 1:** Content selected for dissemination activities

Information shared with all audiences A brief overview of the background and methods of the research study Sample characteristics Summary of the key study findings ∘ Meanings and descriptions of fatigue from the perspectives of young people with SCD in Ghana ∘ Biological and social causes of SCD‐related fatigue ∘ Developmental (physical, emotional, cognitive and social) significance of SCD‐related fatigue for young people with SCD in Ghana ∘ Perceptions of sources of support and support needs for SCD‐related fatigue ∘ Demographic‐based differences in experiences
Additional information tailored to healthcare professionals and charity leads/workers
Parents' impressions of care and support provided by healthcare professionals to them and their adolescent children Parents' recommendations for research, practice and policy change Parents' reported support needs

Abbreviation: SCD, sickle cell disease.

The dissemination project goals and related activities are summarized in Table 2. Between April and July 2021, the two dissemination workshops were planned, organized and conducted. Fifty participants were engaged with the research outcomes, 28 in the parents' workshop and 22 in the health professional and charity lead/volunteer workshops. The healthcare professional participants included two paediatric haematologists, two paediatric medical residents, ten nurses, a pharmacist and a psychologist.

**Table 2:** Dissemination goals and activities

Goals	Activities
Communicate key research findings	Select relevant findings for the target audience Develop varied presentation methods: ∘ Reader‐friendly research reports ∘ PowerPoint presentations
Engage multiple stakeholders to discuss the study findings from diverse perspectives and elicit concrete recommendations for improving care and support for CYP with SCD in Ghana, specific for fatigue	Identify key stakeholders Facilitate attendance of stakeholders at two workshops Brainstorming session
Share feedback and recommendations with all stakeholders	Develop a summary of the brainstorming sessions and resultant recommendations for all targeted stakeholders

Abbreviations: CYP, children and young people; SCD, sickle cell disease.

Communication modes and techniques for enhancing knowledge transfer include presenting information that chimes with experiential knowledge^9^ and making the implications of the findings more salient.^29^ There is advocacy for broad and interactive models of knowledge transfer with activities focused on process and product, emphasizing the crucial elements of reciprocity and exchange between producers and users of knowledge.^31^ Consequently, the primary intent of this project was to move beyond passive, one‐way dissemination of research findings by engaging the stakeholders to discuss their impressions of the study findings and ultimately to support them in developing recommendations for research, policy and practice that they could take forward in their roles as champions of better care and support for CYP with SCD. The two workshops created opportunities for the individual stakeholder groups to meet to debate up‐to‐date evidence and engage with and interpret the research findings based on their shared social constructs, such as beliefs, values, culture and norms. Because knowledge is embedded in social relations,^32^, ^33^, ^34^ this enabled the movement of knowledge despite differences in individual participants' level of experiential knowledge.^9^ Another key approach to knowledge transfer is the understanding that research findings must be *translated* into information that is accessible and meaningful to knowledge users.^35^ Thus, reader‐friendly research reports summarizing the selected findings in versions tailored to parent versus health professional/charity audiences were created in English (Ghana's official language). Although Twi is the commonly spoken Ghanaian language, it is rarely read or written in Ghana.

The two and a half‐hour workshops with the two stakeholder groups were structured interactively to discuss the study findings from different viewpoints. It was intended that this would kindle strategies for improving care and support for CYP with SCD in Ghana. Sessions were organized into four distinct activities: (1) 15‐min prepresentation Q&A about SCD, (2) 45‐min presentation of the key study findings, (3) 30‐min discussion of presented research findings and broader issues beyond the research and (4) 45‐min group ‘brainstorming’ of priorities and recommendations for improving fatigue‐specific care and support for CYP in Ghana and recommendations for future research. The parents' workshop was facilitated in the Twi language and the health professional/charity worker workshop in English. The study findings were presented in an informal format, with the reader‐friendly research reports distributed to all participants to follow as a guide. Presentations were immediately followed by invitations for comments, reactions and ideas from participants.

Different interpretations of the study findings based on participants' experiences and viewpoints were encouraged to contribute new layers of understanding to the original findings. For example, the original study revealed that the young people felt their complaints of fatigue were dismissed by others (including parents and health professionals) and treated as illegitimate. While the adolescents in the study associated these negative social reactions with the invisibility of the symptom, their ‘normative appearance’ and young age,^16^, ^18^ the workshop participants offered several alternate explanations for these observations. These included a lack of understanding of the extent of fatigue's impact on young people with SCD, which some workshop participants associated with the commonality of the experience of tiredness described as ‘fatigue’ in the general population; lack of recognition of the symptom in clinical treatment guidelines due to the absence of evidence‐based SCD‐related fatigue‐specific assessment tools, treatment/support plans and interventions and pragmatic decisions to focus limited resources on acute life‐threatening and objective manifestations of SCD‐like pain.

A brainstorming session then followed the discussions to identify concrete strategies for addressing the issues highlighted by the research findings and the workshop participants' experiences and viewpoints. In light of the study findings, participants were asked to identify barriers and facilitators to providing fatigue‐related care and support for young people with SCD in Ghana based on their experiences and to suggest potential strategies for addressing these. While the first author led all the project workshops and dissemination activities, four facilitators from SCAGH recorded the group's ideas on flip charts. The workshop audiences were grouped into five‐member groups for the brainstorming session and then asked to decide which three ideas should be prioritized for concerted action by all the targeted stakeholders. A written summary of the brainstorming sessions and a summary of the recommendations were subsequently provided to the parents, SCAGH leads and clinical partners.

Following the activities, the participants were asked to evaluate the workshops. The research team developed the evaluation form used to collect participants' feedback regarding the quality of the presentations, the relevance of the information, the likelihood of knowledge usage and the overall usefulness of the workshop. It comprised 13 items: nine closed‐ended questions (on a 5‐point Likert scale from ‘strongly agree’ to ‘strongly disagree’) and four open‐ended questions (see Table 3). The form was tailored for each stakeholder group. Electronic versions of the evaluation form were used. Participants were supported to access the form on their mobile devices, and those without mobile devices were provided with tablets. Participants with literacy limitations were helped by the workshop facilitators to complete the forms. Participants were invited for a short postworkshop interview if they felt they had additional feedback to provide.

**Table 3:** Workshop evaluation (scored on a 5‐point scale from ‘strongly agree’ to ‘strongly disagree’: 5 = *strongly agree*, 4 = *agree*, 3 = *neutral*, 2 = *disagree*, and 1 = *strongly disagree*)

Items	Percentage of participants (score range)	
	Parent workshop (*N* = 23)	Healthcare professional/charity leads/volunteer workshop (*N* = 17)
Closed‐ended		
What I learned today will be useful to support my child or for my work	91.3% (4–5)	100% (4–5)
The workshop was paced well, with room for discussion	91.3% (4–5)	100% (4–5)
The materials were clear and useful	91.3% (4–5)	100% (4–5)
Discussions were useful	95.7% (4–5)	100% (4–5)
Questions were answered well	91.3% (4–5)	100% (4–5)
I am likely to put what I've learned into practice	95.7% (4–5)	100% (4–5)
Overall, I enjoyed the workshop	95.6% (4–5)	100% (4–5)
How likely are you to recommend a similar workshop to another parent or colleague?	91.3% (4–5)	94.1% (4–5)
Can we contact you in the future to assess whether what you have learned today helped you achieve impact?	100% (5)	100% (5)
Open‐ended		
What do you think should be changed in the future?		
What did you find most useful?		
What will you do differently or put into practice due to what you learned today?		
Any comments?		

Following knowledge transfer/sharing, identifying evidence of knowledge usage is an essential aspect of knowledge exchange and research impact. However, funding limitations can sometimes make this challenging. Our funding and project timeline limits meant impact evaluation of the knowledge use post the knowledge transfer activities could not be built into the project. Therefore, the project's main aim was knowledge transfer, assessment of knowledge acceptability and change in knowledge to help address a significant knowledge gap. Nonetheless, 6 months following the workshops, we sent a three‐item questionnaire (two closed‐ and one open‐ended question) to workshop participants who consented for us to contact them in the future regarding knowledge usage and benefits. Although all the 50 participants who attended the workshop agreed for us to contact them in the future, only 22 provided an email address and thus, were sent a questionnaire. The other contact details provided were mobile numbers.

The quantitative data from the immediate postworkshop survey were analysed using Microsoft Excel and summarized using descriptive statistics. Responses to the open‐ended questions from the immediate postworkshop evaluation and the 6‐month follow‐up survey were analysed using the content analysis approach. This comprised a search for meaningful segments (thematic categories) and units (subthemes). Data analysis was conducted by the first author and checked for consistency and accuracy by the second author.

## 3 FINDINGS

### 3.1 Acceptability and satisfaction

In total, 40 participants (23 from the parent workshop and 17 from the healthcare professional workshop) completed the evaluation forms (see Table 3). Almost 96% of the parents and all healthcare professional/charity lead/volunteer participants found the dissemination work useful, and 91% of the parents and 94% of the healthcare professionals/charity lead/volunteers would recommend similar dissemination work to others. The dissemination activities evaluated highly on statements on whether the workshops were well‐paced, enjoyable, materials were clear and useful, discussions were useful and whether questions were answered well (see Table 3). The statements ‘I am likely to put what I've learned into practice’ and ‘What I learned today will be useful for supporting my child or work’ had more than 90% of the participants in each workshop group strongly agreeing. Sections were included on the forms for participants to indicate their reasons for a nonaffirmative response to the evaluation statements. However, none of the respondents who disagreed or strongly disagreed with statements indicated their reasons.

Based on the open‐ended responses, the most valued parts of the workshops were: the presentation of the research findings, which participants felt brought out real‐life experiences (mentioned by all the participants); the ‘brainstorming’ sessions (mentioned by all the participants) and the reader‐friendly summary of the research findings provided (mentioned by 35 participants). Regarding what they would do differently due to what they have learned, all the participants highlighted giving attention to fatigue in the care/support provided to their children or patients/clients and involving the new knowledge in their educational and advocacy work. Some healthcare professionals and charity leads/volunteers noted in open responses that they would educate other colleagues who were not present on fatigue in SCD (mentioned by 15 participants).

Based on their positive experiences, all the participants included the need for similar research dissemination activities in their answers to the question: ‘Is there anything you think should be changed in the future?’ Indeed, all the participating parents and charity leads/volunteers commented that the dissemination workshop was their first opportunity to be engaged with research evidence based on the experiences and voices of CYP with SCD in Ghana, suggesting that knowledge exchange among researchers and community stakeholders that grows out of academic research is still a new concept in Ghana. The workshops also helped clarify and increase SCD‐related fatigue's legitimacy by sensitizing key (family, community and clinical) stakeholders to the extent of fatigue's impact on adolescence. In addition, those who attended the dissemination project workshops were supported to identify strategies and action points to champion practice and policy change to improve the care and support provided to CYP with SCD in Ghana. Indeed, the dissemination project stimulated discussions about how the research findings could best become part of SCD care for CYP that supports and improves the quality of care for them, their families and other care partners.

More broadly, the main goal of this dissemination project was to communicate and discuss the research findings as a springboard for highlighting the need for more attention to be paid to fatigue to facilitate the development of optimal care and support reflecting the priorities of adolescents with SCD in Ghana. Each workshop resulted in a consensus among the stakeholders regarding where future energies should be best directed. The joint priorities selected by the workshop participants were: (1) active acknowledgement of fatigue as a legitimate and significant symptom by all stakeholders; (2) inclusion of fatigue information in all forms of educational and advocacy programmes on SCD; (3) development of informational resources on SCD‐related fatigue tailored for schools, healthcare providers and families; (4) inclusion of fatigue as a primary outcome measure for future interventions and (5) conducting fatigue‐focused future research.

Following the workshop, one paediatric haematologist summarized their learning and action plans as follows:

The workshop has been very insightful that has brought to our attention that fatigue is one of the main symptoms that disturb our patients with SCD. From this workshop, we have been able to get feedback from our young patients … and one of the main highlights is that even though we know that the disease has an adverse effect on our patients' quality of life, we now know that fatigue is one of the main reasons why they don't have a good quality of life. So, … one of the things I'm taking back is that in assessing our patients, aside from asking them about pain, dizziness and palpitations and all the other signs that stem from the low baseline haemoglobin they have, I think one of the things we'll add to our clinical assessment will be fatigue and possibly grade the level of fatigue and also use it as a primary outcome for our interventions. Another thing I'd like to say is that now I know that fatigue is one of the reasons why patients with SCD are stigmatized. Even as a healthcare provider, I didn't really know the impact of fatigue on my patients with SCD. So, when it comes to advocacy, we are now going to create more awareness; let people know that aside from the fact that people with SCD have pains, they also have fatigue. If you don't know about it, you may think that they are lazy; you may single them out and stigmatize them. So, we will create more public awareness concerning fatigue's effects on SCD patients. And also, in our future research, we'd do a general survey on the impact of fatigue on our patients that attend our clinic.

### 3.2 Knowledge usage

All the participants (10 healthcare providers, 8 charity workers and 4 parents) we contacted 6 months post the knowledge transfer workshops completed the questionnaire and provided evidence supporting knowledge application and benefits. Knowledge usage was evident at the family, charity and healthcare team levels, indicating actions towards the joint priorities for practice and policy identified during the workshops. No response was reported regarding policy impact and/or health system‐wide application. This was unsurprising due to the lack of active national standards/recommendations/guidelines for SCD in Ghana and the significant time lags in the health research translation process.^36^ All the respondents reported positive practice outcomes to the open‐ended question, ‘what are you doing differently or have put into practice as a result of what you learned during the workshops?’. The main themes in the responses were *targeted and public education on SCD* and *working together with the child with SCD*.

#### 3.2.1 Targeted and public education on SCD

The commonly applied usage of the knowledge disseminated during the workshops was in educational and advocacy work. This was evident in the responses from healthcare professionals and charity workers. They referred to incorporating fatigue and its impact in their educational and advocacy activities involving healthcare professionals, religious leaders, parents and families and the wider society. They highlighted using the knowledge to address some of the myths and misconceptions about SCD that fuel stigmatization of the illness and those living with it (see Table 4).

**Table 4:** Knowledge usage themes and quotes

Theme	Quotes
Targeted and public education on SCD	Now when I sit on the radio to talk about sickle cell or give talks in hospitals, churches, mosques, schools and community centres about the disease, I talk about fatigue. At first, I focused only on the superstitious myths, stigma and pains. Now, I include fatigue because I understood how fatigue leads to stigma at the workshop. (Charity worker 6)
	We are now including fatigue in our clinical discussions and patient and family education as a team. We are highlighting its importance and effects on the children to create more awareness among our medical and nursing staff and our patients and their families. I also use it in my teaching of medical students during their paediatric placements. We now include fatigue in the medical reports we write to schools regarding our patients as a department. At first, we used to only focus on pains and write things like they were not very strong and should be exempted from physical activities. We know from the workshop that these things we write are wrong because they encourage stigma and exclusion. Now, we indicate that they get tired easily and need to be allowed more rest breaks when they need them. We are working on a small booklet for teachers about SCD, and we are going to include fatigue and its impact. (Healthcare professional 4)
Working together with the child with SCD	After the workshop, I talked to my husband, and we had a conversation with our daughter about her tiredness. It opened our eyes to a lot of the difficulties she was having. She was missing school most of the time because she was too tired from school activities and her chores at home. We have now developed a timetable about chores with her and gotten her brothers to help more in the house. She's now not missing school as much as she used to. We will talk with her teachers about her tiredness and how we can all help her during the next PTA meeting. (Parent 2)
	Involving fatigue in my consultations has opened up a new way of engaging the children. I used to struggle to engage them in a discussion during their consultations. Since I learned fatigue is an everyday experience and part of their day‐to‐day activities at the workshop, I am using it to get them to talk about the difficulties they face due to the condition and the support they need. I think there's more trust now because I'm asking the right questions and paying attention to the issues important to them. (Healthcare professional 8)

Abbreviation: SCD, sickle cell disease.

#### 3.2.2 Working together with the child with SCD

Some respondents also wrote about how the knowledge has opened an opportunity for working together with children with SCD by using fatigue to open dialogue. Some reported how this engagement has led to better understanding and collaboration with the children they care for and support. Increased insight and engagement are prerequisites for delivering empathic, sensitive and tailored care/support, which was evident in some of the reports (see Table 4).

## 4 FUTURE PLANS

The importance of continued collaboration to embed the research findings in the services and support offered to CYP is central to achieving research impact. Consequently, discussions were had with the SCAGH leads and clinical partners following the workshops to identify additional opportunities for the continued usage of the research findings to help concentrate their shared priorities and efforts. Based on the research findings, they identified the need for varied educational resources, for example, videos, animations, leaflets, posters and booklets, to support their educational and advocacy work. We agreed to collaborate in sourcing additional funding to develop such resources in the near future.

## 5 CONCLUSION

The research dissemination project described here was underpinned by an inclusive, reciprocal approach to knowledge transfer to help overcome traditional barriers between knowledge producers and users of research information and generate synergy for collaborative practice change. Engaging multiple stakeholders to discuss the research findings and their related practice, policy and research implications enabled the research team to reinterpret some research findings within a broader context and incorporate new insights. In turn, stakeholders were motivated to take ownership of the research findings and to contribute valuable and actionable recommendations for improving care and support for CYP with SCD in Ghana, thereby stimulating action for evidence‐based practice and policy change.

The timing of the dissemination project was key to its success. Undertaking the project in the month of World Sickle Cell Awareness meant that key stakeholders were already energized and mobilized for educational and advocacy work. Funding which meant that there was no cost to the stakeholders in Ghana, also contributed to a receptive climate for the workshops. However, several limitations of this project are noted. Evaluation of the project activities relied on participant self‐report and did not use previously validated tools. Nonetheless, due to the diverse literacy levels among participants, there was no appropriate pre‐existing tool. Since the funding was short‐term, the project also lacked resources and mechanisms for a robust determination of the impact of the dissemination activities on actual care and support practices and outcomes. Nevertheless, all the workshop participants agreed to be contacted in the future to assess whether what they learned helped achieve subsequent impacts. Some have already been followed up to share information regarding their short‐term usage of the knowledge and the benefits therein, as we have reported here.

This article describes a model of developing, planning and disseminating research findings in an LMIC that links research dissemination activities to broader clinical and community goals for improving care and support for CYP with SCD in Ghana. The partnership, planning, activities and outcomes described underscore the essential benefits of active dissemination activities for developing both practice and research in LMICs. Although limitations of time, funding and global pandemic constrained what could be achieved here, we suggest that future dissemination projects should be geared towards meeting local informational needs by being grounded in local empirical findings and knowledge, rather than untested theoretical propositions, and involve systematic process and outcome evaluation to contribute to the growing field of knowledge transfer, particularly in LMICs. Equally important, actual dissemination activities and outcomes such as those described herein must continue to be documented widely so that all engaged in dissemination activities in LMICs may benefit from the experience of others.

## CONFLICT OF INTEREST

The authors have no conflict of interest relevant to this article to disclose.

## Data Availability

All relevant data are within the paper.
